# Development of Norelgestromin Dissolving Bilayer Microarray Patches for Sustained Release of Hormonal Contraceptive

**DOI:** 10.3390/pharmaceutics16070946

**Published:** 2024-07-17

**Authors:** Lalitkumar K. Vora, Ismaiel A. Tekko, Fabiana Volpe Zanutto, Akmal Sabri, Robert K. M. Choy, Jessica Mistilis, Priscilla Kwarteng, Maggie Kilbourne-Brook, Courtney Jarrahian, Helen O. McCarthy, Ryan F. Donnelly

**Affiliations:** 1 School of Pharmacy, Medical Biology Centre, Queen’s University Belfast, 97 Lisburn Road, Belfast BT9 7BL, UK; l.vora@qub.ac.uk (L.K.V.);; 2 PATH, 2201 Westlake Avenue, Seattle, WA 98121, USA; rchoy@path.org (R.K.M.C.);

**Keywords:** norelgestromin, microarray patch, microneedles, hormonal contraception, intradermal delivery, sustained-release, pregnancy prevention

## Abstract

Microarray patches (MAPs) offer a noninvasive and patient-friendly drug delivery method, suitable for self-administration, which is especially promising for low- and middle-income country settings. This study focuses on the development of dissolving bilayer MAPs loaded with norelgestromin (NGMN) as a first step towards developing a future potential drug delivery system for sustained hormonal contraception. The fabricated MAPs were designed with the appropriate needle lengths to penetrate the stratum corneum, while remaining minimally stimulating to dermal nociceptors. Ex vivo assessments showed that the MAPs delivered an average of 176 ± 60.9 μg of NGMN per MAP into excised neonatal porcine skin, representing 15.3 ± 5.3% of the loaded drug. In vivo pharmacokinetic analysis in Sprague Dawley rats demonstrated a Tmax of 4 h and a Cmax of 67.4 ± 20.1 ng/mL for the MAP-treated group, compared to a Tmax of 1 h and a Cmax of 700 ± 138 ng/mL for the intramuscular (IM) injection group, with a relative bioavailability of approximately 10% for the MAPs. The MAP-treated rats maintained plasma levels sufficient for therapeutic effects for up to 7 days after a single application. These results indicate the potential of NGMN-loaded dissolving bilayer MAPs, with further development focused on extending the release duration and improving bioavailability for prolonged contraceptive effects.

## 1. Introduction

According to recent estimates by the United Nations, approximately 164 million women who want to avoid pregnancy are not using safe, modern contraception [1]. Additionally, nearly 85 percent of these women live in low- and middle-income countries [2]. Almost half of all pregnancies globally (121 million) are unintended, which may lead to significant health, economic, and psychosocial costs. Many factors influence a woman’s ability to use contraception, including societal and health system- and product-related factors [3].

Recent analysis of unmet need revealed that a lack of awareness of and access to contraception are no longer cited as the leading causes of poor contraceptive usage [4]. Additionally, although a range of both short-term and long-acting contraceptive methods exist, high rates of method discontinuation are being observed [5]. Women who have access to contraception may not use or may discontinue use for several reasons, including changes in relationship status or fertility intentions, concerns over side effects, and opposition from others [5]. Thus, the definition of unmet need should include both women who do not have access to appropriate preventive methods and those who are dissatisfied with their current method [6].

Among the reversible contraceptive methods, hormonal drug delivery systems represent the broadest array of products, including multiple types of oral pills, injectables, and transdermal patches, as well as vaginal rings, implants, and hormone-releasing intrauterine devices [1,7]. These products differ in the types of hormones delivered and the dosing regimen. User-initiated methods such as oral pills, transdermal patches, and vaginal rings require daily, weekly, or sometimes monthly dosing. Injectables provide 2 to 3 months of protection, and in most countries require a clinic visit for administration but can also be self-administered. Methods such as intrauterine devices and implants provide long-term protection (3 to 8 years) and require a provider to insert the product [8,9].

Nonetheless, there remain product-specific concerns that may impact patient nonuse, noncompliance, or discontinuation of these methods, which affects their overall success rates. For example, when considering options that require daily use for effective protection, like oral pills, the cost of the medication and strict adherence requirements are common challenges. Concerns regarding other longer-acting methods include cost, injection site pain, the need for frequent clinic visits (for injectables), and the need for surgical procedures to insert and remove implants. Since the effectiveness of user-initiated methods depends on patient compliance and adherence to product use, new drug delivery systems are needed to address user concerns and provide women and girls with increased options of contraception that fit with their needs and lifestyles [10].

Microarray patches (MAPs), also known as microneedle patches, are under development for the delivery of drugs and vaccines, including for the prevention of pregnancy [11,12,13]. Dissolving MAPs, in particular, consist of an array of water-soluble or biodegradable polymers and the drug(s) that dissolve and release their payload upon exposure to interstitial fluid in the skin [14,15]. MAPs are minimally invasive devices that are inserted into the skin through applied pressure, facilitating drug delivery in a way that is typically perceived as less painful than needles and syringes [16,17,18].

Contraceptive MAPs can be formulated for the controlled release of the drug and are considered easy to use [12,19]. Once a user has been trained to apply MAPs correctly, they can potentially be self-applied at home whenever subsequent dosing is required [20]. This is especially beneficial for women and girls in low- and middle-income countries, where there is great need for contraceptive options that do not require frequent health care visits, as access to health facilities and trained health care providers may be limited, particularly in more rural areas.

There are studies in the literature that support the potential feasibility of MAPs as a long-acting contraceptive delivery platform. One study highlighted the possibility of a controlled release of levonorgestrel for 6 months in vitro through the development of a core–shell MAP made from biodegradable poly(lactic-co-glycolic acid) (PLGA) and polylactic acid (PLA) polymers [21]. Another study demonstrated preclinical in vivo success in the development of contraceptive MAPs that can deliver a sustained release of levonorgestrel through the detachment and embedding of biodegradable PLGA and PLA microneedles into the skin for at least 1 month [22]. Other studies have also highlighted the use of other hormonal contraceptive drugs, including etonogestrel and progesterone, as well as biodegradable materials, including silk fibroin, polyvinylpyrrolidone (PVP), polyvinyl alcohol (PVA), and hydroxypropyl cellulose, to fabricate MAPs with a range of other delivery efficiencies and durations of protection [23,24,25].

Considering the current contraceptive MAP landscape, we designed a dissolving bilayer MAP that aimed to deliver an in vivo sustained release of norelgestromin (NGMN) for a 1- to 3-month duration of protection. Feedback on the ideal duration provided by end users in several studies that assessed MAP product preferences and potential acceptability influenced this target; women in several low- and middle-income countries expressed a desire for MAPs that could protect users for up to 1, 3, or 6 months [26,27,28]. Additionally, NGMN was chosen as the candidate drug for this MAP, since it is already marketed in a conventional transdermal patch format, as Mylan Pharmaceuticals’ Xulane^®^ (delivered with ethinyl estradiol), potentially simplifying the regulatory approval process for such a product. However, unlike the Xulane patch, which was designed to be worn and reapplied every week [29], our MAP was designed to be removed after a short application period of 20 min to 1 h, while still aiming to maintain long-term therapeutic effects.

## 2. Materials and Methods

### 2.1. Materials

Norelgestromin was purchased from Toronto Research Chemicals, Inc. (Toronto, ON, Canada). PVA of molecular weight 9 to 10 kDa, 80% hydrolyzed (PVA 10K), and of molecular weight 31 to 50 kDa, 87% to 89% hydrolyzed (PVA 50K), was purchased from Sigma-Aldrich (Dorset, UK). PVP K-29/32 of molecular weight 58 kDa was provided by Ashland (Kidderminster, UK). A liquid silicone elastomer mix was purchased from Nusil Technology (Buckinghamshire, UK). Ultrapure water was obtained from a water purification system (Elga PURELAB DV 25, Veolia Water Systems, Dublin, Ireland). All other chemicals and materials were of analytical reagent grade and supplied by Sigma-Aldrich.

### 2.2. Methods

#### 2.2.1. Fabrication of NGMN-Loaded Bilayer Dissolving MAPs

**Figure 1:**
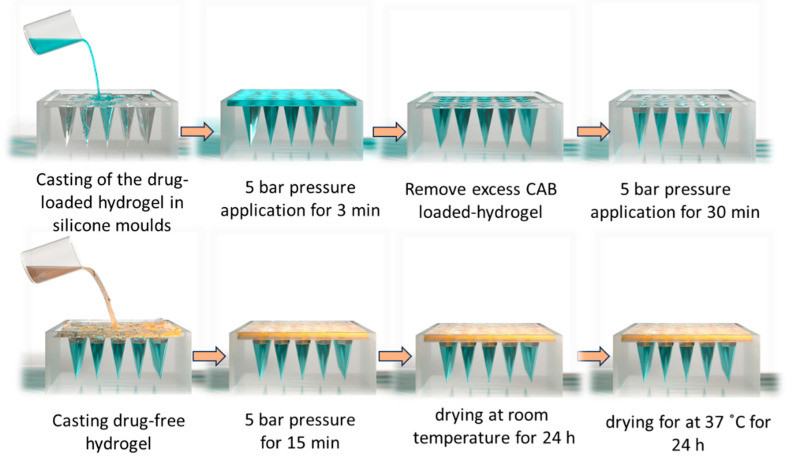
Manufacturing steps of bilayer NGMN microneedle arrays.

The overall manufacturing process is illustrated in the steps below (Figure 1). First, NGMN (100 mg) was mixed with 187.5 mg of PVA 10K (40% *w*/*w*), 187.5 mg of PVP K-29/32 (40% *w*/*w*), and 525 mg of water. This mixture was then homogenized with two metal beads in a TissueLyser LT (QIAGEN^®^, Manchester, UK) at 50 Hz for 30 min to create the uniform drug suspension. The first layer was cast with this NGMN mixture into MAP molds (needle density of 16 × 16, cuboidal needles 900 μm in height, column part 300 μm, and pyramidal tip part 600 μm in height, with a base width of 300 μm and interspacing of 100 μm). Subsequently, MAP molds were placed in a positive pressure chamber for 3 min at a pressure of 5 bars, after which excess mixture was scraped. The molds were then placed in the positive pressure chamber for 30 min before being left to dry at an ambient temperature for 24 h. Afterward, the drug-free second layer was cast with a mixture of PVP K-29/32 (20% *w*/*w*) and PVA 50K (15% *w*/*w*) hydrogels (50:50 *w*/*w*). The MAP molds were then placed in a positive pressure chamber for 15 min at 5 bars. Finally, the arrays were dried at room temperature for 24 h and then at 37 °C for 24 h.

#### 2.2.2. Physical Characterization

The NGMN microneedles were visualized with the help of digital and scanning electron microscopes (SEM). The stereo microscope, specifically the EMZ4™ model from Leica Microsystems (Milton Keynes, UK), was used to obtain three-dimensional visualizations of the microneedles. The SEM was used to achieve high-resolution images of the microneedles, offering detailed insights into their surface morphology and structural features. A High-Resolution Environmental SEM (Quanta FEG 250, FEI, Hillsboro, OR, USA) was used for the detailed characterization of the microneedles. This SEM was operated at acceleration voltages ranging from 10 to 20 kV, allowing us to achieve an optimal resolution and the depth of field necessary for capturing the fine structural details of the microneedles. The use of high chamber pressure was particularly beneficial in maintaining the integrity of the samples by reducing the charging effects, which is crucial for the accurate imaging of non-conductive and hydrated specimens. This feature eliminated the need for conductive coatings, thus preserving the natural state of the microneedles. The Quanta FEG 250 proved ideal for the detailed examination of the microneedles’ pyramidal tips, enabling us to gather valuable insights into their sharpness and potential penetration efficiency, which are critical parameters for their effectiveness in transdermal drug delivery applications.

The compression properties of the microneedles were detected with a TA.XT2 texture analyzer (Stable Microsystem, Haslemere, UK) in compression mode, as previously reported [11,16]. The initial heights of the microneedles were first measured using a stereo microscope. Subsequently, microneedle arrays were affixed to the movable cylindrical probe of the texture analyzer using double-sided adhesive tape and forced by the test station against a flat aluminum block at a rate of 0.5 mm/s for 30 s and a force of 32 N (0.088 N/needle). Pretest and post-test speeds were specified as 1 mm/s, and the trigger force was specified as 0.049 N. Microneedle heights were determined again using the stereo microscope, and the percentage reduction in height following the application of the axial compression load was calculated using Equation (1).

%Height reduction = (Original height − New height)/(Original height) × 100%(1)

The insertion properties of the microneedle arrays were analyzed with the same setup as the mechanical strength test by lowering the microneedle arrays onto a stack of eight layers of Parafilm M^®^ (Bemis, Inc., Soignies, Belgium).

#### 2.2.3. Analytical Method

An HPLC method was developed and validated to quantify the NGMN in the conditions for routine analysis. In total, 10 mg of NGMN was weighed and dissolved in 2 mL of methanol, then diluted in range of 0.5–50 µg/mL for the NGMN calibration curve, covering the detected concentrations in the in vitro study. Analyses of drug samples were performed on the Agilent 1260 Infinity II LC system (Agilent Technologies UK Ltd., Stockport, UK). The separation and quantification of the drug was accomplished using a Symmetry^®^ C18 column (4.6 mm × 150 mm, 5 µm) (Waters; Milford, MA, USA) with an isocratic elution. The eluent consisted of acetonitrile (mobile phase A) and water containing 0.1% *v*/*v* triethylamine adjusted to pH 6.6 (mobile phase B) with a ratio of 35:65 (*v*/*v*). The flow rate was set to 0.8 mL/min. The injection volume was 50 µL, and the column temperature was maintained at 40 °C. The detection was performed at a wavelength of 254 nm. The total run time for the analysis was 10 min. The final method had an R^2^ value of 1 and a limit of quantitation (LOQ) of 0.25 µg/mL. The method had an inter-day variability of 1.62%, and an intra-day variability of 0.83%, indicating good precision. Furthermore, the method exhibited stability over 3 days at room temperature with an assay percentage of 98.58 ± 0.85%, indicating the method’s reliability and robustness for NGMN detection.

#### 2.2.4. Drug Content and Deposition Study

The drug content of the microneedle arrays was determined by dissolving them in 5 mL of deionized water and stirring them at 200 rpm for 30 min, and then transferring 100 μL of the resultant suspension into 1.5 mL Eppendorf Tubes^®^ (Eppendorf SE, Hamburg, Germany). The suspension was then diluted with 900 μL of acetonitrile to dissolve the NGMN and ensure the precipitation of the PVA and PVP polymers, and then vortexed for 5 min. Subsequently, the mixture was centrifuged at 14,800 rpm for 15 min. The amount of NGMN in the resulting supernatants was analyzed using the high-performance liquid chromatography–ultraviolet method.

NGMN skin deposition was studied using full-thickness excised and shaved neonatal porcine skin. The NGMN microneedle array was inserted into the skin using thumb pressure for 30 s and secured using a 20 g stainless steel weight for 24 h, after which the drug was extracted from the skin and quantified.

#### 2.2.5. In Vivo Study

The Queen’s University Belfast Committee of the Biological Services Unit approved the animal studies. All researchers conducting the animal work had obtained personal licenses from the UK Home Office. Female Sprague Dawley rats (Charles River Laboratories, Harlow, UK), 8 to 10 weeks of age (weight: 244 g ± 12 g for the intramuscular (IM) group and 237 g ± 6 g for the MAP group), were used to compare the pharmacokinetics of the two different drug administration methods—IM injection and MAP—with a focus on their potential for a sustained release profile. The rats were acclimatized to the animal housing conditions for 1 week prior to the experiment and separated into two cohorts of six rats each. Table 1 presents the experimental setup.

For MAP application, the animals were anesthetized using gas anesthesia with 5% isoflurane in oxygen at a flow rate of 2 L/min. Maintenance anesthesia was achieved by reducing the isoflurane concentration to 2.5% *v*/*v*, with a flow rate of 2 L/min. First, the back of each animal (i.e., the intended site of application) was shaved using electric hair clippers (Remington Co., London, UK) to minimize the interference of fur during MAP application. Afterwards, the remaining fur was removed by applying a depilatory cream (Boots Smooth Care Hair Removal Cream for sensitive skin, Boots, Nottingham, UK). Following this, the rats were left for a 24 h period to allow their skin to recover and to ensure the complete restoration of the skin’s barrier function before MAP application. On the following day, the MAPs were administered to the rats. The MAPs were secured in place using Microfoam™ Surgical Tape (3M, St. Paul, MN, USA), which was additionally secured using 3M Tegaderm™ film and kinesiology tape for 24 h.

To prepare the NGMN suspension for IM injection, 50 mg of NGMN was dispersed in 1 mL of 2% PVA (stabilizer) and homogenized for 30 min using a TissueLyser (Qiagen, Hilden, Germany) at 50 rpm. This prepared NGMN suspension was IM injected at a dose of 2 mg (40 µL) of NGMN per animal.

Blood samples were collected in 1.5 mL pre-heparinized microtubes over 2 weeks via tail vein bleeds following NGMN administration (either IM or using MAPs). After collection, the samples were centrifuged immediately at 2200× *g* for 10 min at 4 °C, and the plasma was collected and stored at −20 °C until further analysis with high-performance liquid chromatography–mass spectrometry.

## 3. Results and Discussion

### 3.1. Microscopy and Mechanical Characterization

**Figure 2:**
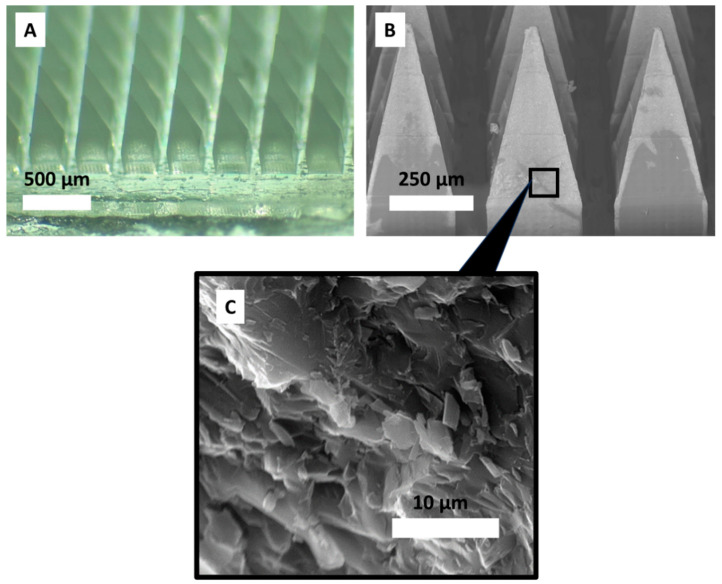
(**A**) Digital microscopy image. Representative SEM images of tip-loaded microneedles containing NGMN, showing (**B**) a fully formed microneedle array and (**C**) a needle tip matrix.

The microscopy images below show that the bilayer microneedles used in this study had sharp tips and smooth surfaces (Figure 2A), implying the successful use of the manufacturing method to produce consistently defect-free bilayer structures. This was also confirmed using the SEM (Figure 2B), which showed that the NGMN microneedle shafts appeared to have a smooth surface. Drug particulates distributed within the microneedle polymeric matrix can be seen in Figure 2C.

**Figure 3:**
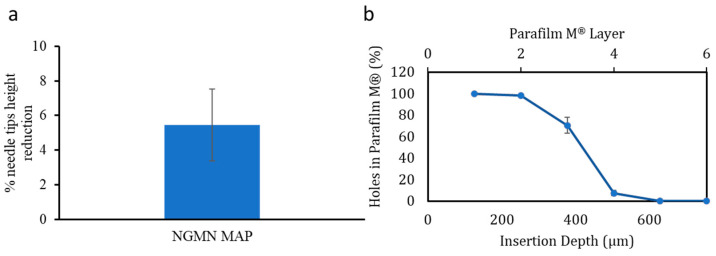
(**a**) Assessment of mechanical properties of NGMN MAPs using a texture analyzer, measured by the percentage of height reduction in the MAP shaft under a 32 N compression force against an aluminum block (mean ± standard deviation [SD], *n* = 3). (**b**) Evaluation of NGMN microneedle penetration in eight Parafilm M layers as an artificial skin model, indicating the percentage of holes and corresponding insertion depths achieved with a 32 N force (mean ± SD, *n* = 3).

For MAPs to successfully deliver the payload into the patient following skin application, the system must possess sufficient mechanical strength to withstand the application force to enable the insertion of the microneedles into the skin. NGMN microneedle arrays were tested for mechanical strength via a height reduction test [30,31]. In the current work, the NGMN microneedle array displayed a height reduction of 5.4 ± 2% (shown in Figure 3a). Based on our previous work, this height reduction indicates this microneedle array exhibited sufficient mechanical robustness to withstand the application force during insertion without buckling or fracturing on the skin surface [11,14].

Further mechanical characterization was conducted by evaluating the insertion profile of the NGMN MAP using Parafilm M as an in vitro skin model [32]. The mean thickness of a Parafilm M layer is 126 ± 7 µm. The MAP insertion studies found that microneedles inserted within the third and fourth layers of the skin simulant model resulted in an overall insertion depth of approximately 378 µm (shown in Figure 3b). This insertion depth would suggest the MAP could be adequately inserted into the dermal layer of the skin [33]. This would be the targeted insertion depth needed to deliver the payload, as the dermal layer is rich in microcirculation for carrying the released payload into systemic circulation.

### 3.2. Drug Loading and Skin Deposition

Following an evaluation of the mechanical strength and insertion capabilities of the patches, a skin deposition study was conducted to evaluate the amount of drug that can be successfully delivered into the skin, as well as the delivery efficiency of the formulation. In general, the formulated patches had an overall drug load of 1150 ± 177 µg. The ex vivo skin deposition of the NGMN MAP was found to be 176 ± 60.9 μg per MAP, equating to a deposition of 15.3 ± 5.3% of drug payload in excised neonatal porcine skin, as shown in Table 2. This low delivery efficiency may be attributed to the micronized hydrophobic nature of the drug, indicating a less favorable deposition into the water-rich dermis [34]. The physiochemical properties of the active pharmaceutical ingredient and particle size have a critical impact on the delivery efficiency of this dissolving MAP design.

For example, in a previous research study assessing contraceptive delivery via microneedles, Nestorone^®^ nanosuspension–loaded microneedles demonstrated a high drug deposition at 904 µg (about 40% of the loaded dose of 2260 µg). This higher deposition could be the result of the nanosuspension formulation, which likely enhanced the solubility and bioavailability of the drug within the skin layers [35]. In contrast, the delivery efficiency of microneedles loaded with the micronized form of Nesterone was comparatively low under similar conditions, with the highest deposition recorded at 504 µg, representing about 25% of the loaded dose (2160 µg). This reduction in the deposition efficiency of the Nestorone nanosuspension relative to the micronized form of Nestorone could be attributed to differences in the formulation and physical state of the drug within the microneedles.

The low delivery efficiency observed in our skin deposition studies can be attributed to the physicochemical properties of the drug. The hydrophobic nature of NGMN, which exhibits a logP of 4.04, likely reduced the dissolution of the drug into the skin, culminating in a reduction in payload delivery. In addition, the high level of drug loading (40% *w*/*w*) in this study may have contributed to the incomplete dissolution of the microneedles within the 24 h timeframe of the experiment, resulting in some of the matrix being dislodged during patch removal, and thus reducing delivery efficiency.

### 3.3. In Vivo Study Evaluation

**Figure 4:**
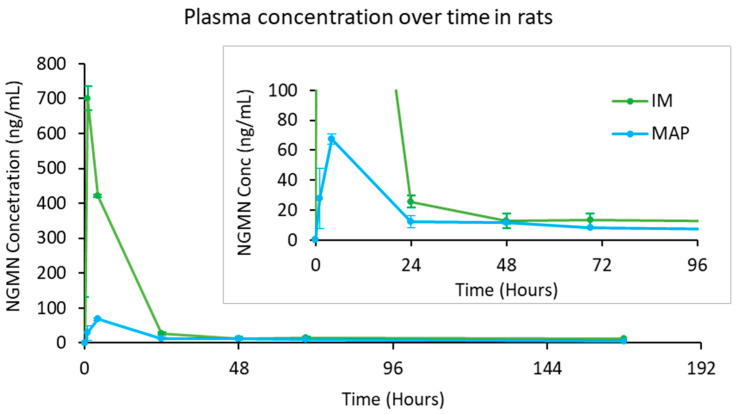
Pharmacokinetic profile of NGMN in Sprague Dawley rats following administration (2 mg NGMN suspension per rat) by IM injection or by applying four dissolving NGMN MAPs. Data are reported as the means ± SDs (*n* ≥ 3).

Following the in vitro characterization of the fabricated NGMN-loaded MAP, the formulation was evaluated further in vivo. The pharmacokinetics of NGMN following administration via MAP or IM injection are shown in Figure 4 and Table 3. The rats that received NGMN by IM injection reached peak plasma concentrations of the drug (C_max_ of 700 ± 138 ng/mL) at 1 h (T_max_). After that, NGMN levels decreased rapidly to approximately 25.7 ± 12.2 ng/mL on day 1 and then declined gradually to reach their lowest concentration of 11.2 ng/mL on day 7. By day 14, NGMN was below the limit of quantification. In contrast, the rats that received the drug via dissolving MAP exhibited a steady increase in NGMN concentration in the plasma that reached a C_max_ of 67.4 ± 20.1 ng/mL at 4 h. Afterwards, the drug plasma levels decreased to approximately 5 ± 0.638 ng/mL on day 7 and went below the limit of quantification after day 14.

The IM group had a much more rapid C_max_ and a higher area under the curve (AUC) relative to the MAP-treated group. This is due to higher dose administered creating a large diffusion gradient and the administration of the drug directly in the capillary-rich muscle tissue, which enables dissolution and the diffusion of the drug into the systemic circulation. This is further enhanced by the fact that muscle tissue, due to its intrinsically high metabolic rate, has a much denser network of blood capillaries relative to skin tissue [36]. This would provide more circulatory surface area for NGMN to diffuse from the injection site and into the systemic circulation. In contrast, the MAP treatment group had a more delayed T_max_ relative to the IM treatment group. This may be attributed to the fact that when NGMN was deposited into the skin via MAP application, the drug needed to first dissolve from the PVP/PVA matrix to then diffuse through the skin tissue before reaching dermal circulation.

The C_max_ and AUC for the MAP-treated group were much lower (*p* < 0.05) than those for the IM treatment group. This may be attributed to the lower estimated dose delivered (approximately 15%) or the lower MAP bioavailability (10.36%) for the MAP-treated group relative to the IM group. This finding is consistent with the NGMN ex vivo skin deposition studies, which suggest incomplete drug delivery from the MAPs. Unlike the complete delivery achieved with IM injections, an incomplete delivery of drugs loaded within the MAP tips was observed in previous studies [37]. The lower dose delivery could be caused by the “bed-of-nails” effect, which could be mitigated by further optimizing the drug load towards the microneedle tips, increasing the dissolution of the microneedle matrix, or optimizing the release of the microneedles from the patch backing. The bioavailability of drugs delivered transdermally via dissolving MAPs tends to decrease with increasing logP values [38]. The logP values of NGMN is 4, indicating a low bioavailability in transdermal absorption when administered by dissolving MAPs due to the hydrophobic nature of the micronized drug. This results in more undissolved drug remaining in the skin tissue. Therefore, enhancing solubility through techniques such as nanosuspension [35] or cyclodextrin complexation [39] may mitigate the effects of high logP values, potentially improving overall drug bioavailability.

The mechanism of MAP-based transdermal delivery can be broken down into three main steps—(i) application, (ii) dissolution, and (iii) diffusion. First, the bilayer design of the MAP allows for the initial penetration of the microneedle across the lipid-rich stratum corneum, enabling the deposition of the drug-loaded polymeric matrix into the dermis. The interstitial fluid in the dermis encounters the fast-dissolving PVA and PVP matrix of the MAP backing, leading to its rapid dissolution. The drug-loaded tips are left in the skin and exposed to the interstitial fluid. The dissolution of the drug from the surface of the MAP tips then results in the initial burst release of NGMN. The inner portion of the tips form a drug depot in the skin, allowing for the removal of the backing after the wear time.

The release of the NGMN from the MAP will result in the formation of a localized region of high drug concentration. This would then promote the NGMN to undergo Fickian diffusion from the site of administration to the surrounding capillary bed, where the drug would diffuse into the micro-capillaries, resulting in transdermal delivery.

The micronized form of NGMN used in the microneedle impacts its solubility and deposition. Smaller particle sizes generally enhance the dissolution rate due to the larger surface to volume ratio. The initial phase of the drug release from the MAP may be attributed to the drug dissolution that occurs on the surface of the microneedle, as well as the dissolution of smaller NGMN particles. In contrast, larger particles, as well as the payload located in the upper portion of the polymeric matrix, will undergo a much more delayed release that may result in a lower deposition, due to incomplete dissolution during the application period. Our in vivo studies showed that the NGMN MAPs maintained detectable plasma levels for up to 7 days. This sustained release is attributed to the gradual dissolution of the microneedles and the continuous release of NGMN from the particulate depot into the systemic circulation.

NGMN plasma levels achieved using the MAPs for 7 days exceeded 0.6 to 1.2 ng/mL, which is the target level needed in humans for contraception [40]. In addition, the plasma levels exhibited by MAP-treated rats between days 2 and 7 were similar (*p* > 0.05) to those exhibited by IM-treated rats. The much lower AUC (*p* < 0.05) achieved by the MAP treatment group relative to IM injection would suggest there would be limited systemic exposure to NGMN following patch application.

These findings also align with what was seen previously with Nestorone when delivered via dissolving MAPs in vivo, which found sustained plasma levels for up to 6 days [35]. Other dissolving contraceptive MAP development efforts using levonorgestrel found that alternative formulation approaches, such as the inclusion of PLGA and/or PLA release-retarding polymers, achieved a higher dose delivery efficiency and capability for sustained release for several months [21,41]. To advance towards the development of MAPs capable of meeting women’s needs for long-acting contraception [25,26,27], further research is needed on such strategies for the incorporation of these release-retarding polymers as a polymeric particulate form or as a part of a MAP tip matrix, and to optimize MAP formulations for enhanced drug stability and release kinetics. Future studies should also focus on increasing the bioavailability of contraceptive drugs delivered via MAPs and on addressing the challenge of the incomplete dose delivery observed in our findings. This progression will involve detailed investigations into innovative polymer matrices and comprehensive in vivo studies to ensure long-term therapeutic efficacy and safety.

Despite our comprehensive evaluation of these MAPs at an in vitro and in vivo level, there are several limitations associated with the current work. The use of Sprague Dawley rats as a model for human skin and systemic absorption may not fully replicate the human physiological response to NGMN delivery via MAPs. For instance, the presence of panniculus, a layer of muscle in rat skin but absent in humans, may impact the insertion of MAP into the skin. In addition, rat skin is intrinsically thinner than that of human skin, which may have resulted in an over estimation of microneedle penetration and delivery. This is further compounded by inter-species metabolic differences between rats and humans that could affect the extrapolation of the results.

Also, this study focused on a single application of MAPs. The results might differ with repeated applications, which would be more accurate and representative of actual clinical scenarios. Therefore, future studies ought to investigate the long-term effect after repeated MAP application on the plasma profile of the drug, as this could provide a more comprehensive understanding of the long-term effects and safety of the MAPs. Another apparent limitation of this study was the size of the patch used in this study, which had an area of 0.36 cm^2^, appropriate for insertion into rats. It is most likely that in order to translate this formulation and technology into a clinical setting, a scaling up of these MAPs would be pertinent. This in turn would require the evaluation of these patches from in vivo pharmacokinetic and manufacturability perspectives. In addition, these larger patches would also require end-user evaluation to determine any human factors which would promote or hinder the acceptance or usage of these patches.

## 4. Conclusions

The research findings demonstrate the fabrication of dissolving bilayer MAPs for the delivery of NGMN, evaluating a potential approach to the delivery of hormonal contraception. Microscopic and mechanical characterization confirmed the integrity of the microneedle structures, indicating their suitability for skin penetration. The microneedles exhibited minimal height reductions under compression, ensuring their mechanical robustness during insertion. Ex vivo skin deposition studies revealed that the drug deposition efficiency was low; the in vivo study confirmed this and found that the MAP provided a sustained delivery of the drug over a period of only 7 days. Therefore, improvements would be needed to reach the targeted 1- to 3-month duration of efficacy, such as incorporating polymers that can prolong drug release and enhance the MAP’s drug delivery efficiency.

## Figures and Tables

**Table 1:** Rat cohorts, treatment groups, and doses applied per rat.

	Cohort 1	Cohort 2
Number of rats	6	6
Formulation	IM injection	MAP
Dose applied	40 µL (2 mg)	4 MAPs (1.2 mg/MAP)
Estimated administered dose	2 mg	0.72 mg
Application time	Not applicable	24 h

**Table 2:** Drug content (mg/MAP) and amount of NGMN deposited in skin following MAP application (mean ± SD, *n* ≥ 3).

NGMN MAP	Drug Content/per MAP (µg)	Drug Amount Deposited into the Skin (µg)	Drug Amount Deposited in the Skin (%)
Average	1150 ± 177	176 ± 60.9	15.3 ± 5.3

**Table 3:** The pharmacokinetic parameters of NGMN in Sprague Dawley rats following administration of IM injection or application of four dissolving NGMN MAPs. Data are reported as the means ± SDs, *n* = 6.

Pharmacokinetic Parameter	IM Injection	MAP
Applied dose (mg)	2.0 mg	4.8 mg total in 4 MAPs (1.2 mg/MAP)
Estimated dose administered/rat (mg)	2.0	0.72
Average rat weight (g)	244 ± 12	237 ± 6
T_max_ (h)	1.0	4.0
C_max_ (ng/mL)	700 ± 138	67.4 ± 20
AUC_0–168_ (ng/mL/h)	8623 ± 799	2146 ± 218
Relative % drug found in plasma compared to IM	Not applicable	10.36 ± 1.56

## Data Availability

All data are included in this manuscript and its Supplementary Materials.

## Supplementary Materials

The supporting information can be downloaded at: https://www.mdpi.com/article/10.3390/pharmaceutics16070946/s1.
